# Saturating relationship between phytoplankton growth rate and nutrient concentration explained by macromolecular allocation

**DOI:** 10.1016/j.crmicr.2022.100167

**Published:** 2022-09-21

**Authors:** Jongsun Kim, Gabrielle Armin, Keisuke Inomura

**Affiliations:** a School of Earth, Environmental, and Marine Sciences, University of Texas Rio Grande Valley, Brownsville, TX, USA; b Graduate School of Oceanography, University of Rhode Island, Narragansett, RI, USA

**Keywords:** Monod kinetics, Phytoplankton, Macromolecular allocation, Nutrient, Growth, Protein, Carbohydrate, Lipid, DNA, RNA, Nutrient storage

## Abstract

Phytoplankton account for about a half of photosynthesis in the world, making them a key player in the ecological and biogeochemical systems. One of the key traits of phytoplankton is their growth rate because it indicates their productivity and affects their competitive capability. The saturating relationship between phytoplankton growth rate and environmental nutrient concentration has been widely observed yet the mechanisms behind the relationship remain elusive. Here we use a mechanistic model and metadata of phytoplankton to show that the saturating relationship between growth rate and nitrate concentration can be interpreted by intracellular macromolecular allocation. At low nitrate levels, the diffusive nitrate transport linearly increases with the nitrate concentration, while the internal nitrogen requirement increases with the growth rate, leading to a non-linear increase in the growth rate with nitrate. This increased nitrogen requirement is due to the increased allocation to biosynthetic and photosynthetic molecules. The allocation to these molecules reaches a maximum at high nitrate concentration and the growth rate ceases to increase despite high nitrate availability due to carbon limitation. The produced growth rate and nitrate relationships are consistent with the data of phytoplankton across taxa. Our study provides a macromolecular interpretation of the widely observed growth-nutrient relationship and highlights that the key control of the phytoplankton growth exists within the cell.

## Highlights

• Macromolecular model reproduces widely observed growth rate nutrient relationship.
• The slope of the relationship is affected by the intracellular level of proteins.
• Maximum growth rate is constrained by the macromolecular allocation.

## Graphical abstract

**Figure:**
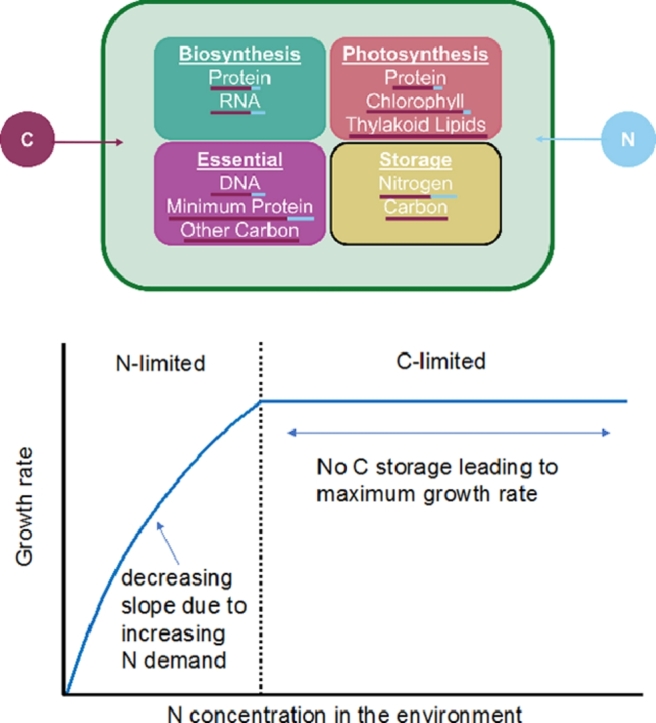

## 1 Introduction

Phytoplankton are at the core of the marine food web, contributing to about 45% of the net primary production globally (Falkowski, 2012; Pierella Karlusich et al., 2020). As a key player in the biological carbon pump, phytoplankton affect primary production, global biogeochemical cycles, and the surrounding marine ecosystem (Falkowski, 2012; Falkowski et al., 1998; Sharoni and Halevy, 2022). They consume carbon (C) and nutrients (i.e., nitrogen (N) and phosphorous (P)) in the ocean to build cellular molecules, resulting in a similar elemental composition in the ocean determined by the Redfield ratio (C:N:*P* = 106:16:1) (Deutsch and Weber, 2012; Falkowski, 2012; Redfield, 1958). Moreover, nutrient supply controls the growth rate, size, and proliferation of phytoplankton and acts as a major limiting factor (Mei et al., 2009; Rhee, 1978; Ward et al., 2017). However, changes to their environment such as eutrophication and climate change, which lead to the change of phytoplankton's elemental composition (Schulhof et al., 2019), can alter the nutrient supply, leading to a variation in cellular elemental ratios and phytoplankton growth rate, which can ultimately change marine organic matter available for other organisms to use (Branco et al., 2018; Martiny et al., 2013; Schulhof et al., 2019). Accordingly, the relationship between nutrient supply, growth rate, and the elemental ratio of phytoplankton is essential to assess marine ecology and global biogeochemical cycles.

**Fig. 1:**
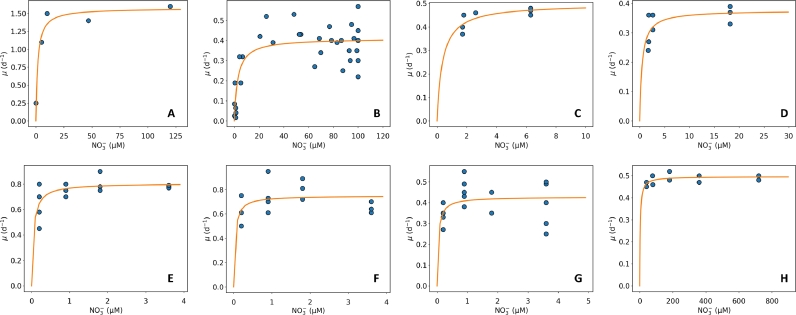
Growth rate vs NO3- concentration with Monod formulation. Dots are data and curves are Monod formulation. (A) *Synechocystis* sp. PCC6803 (Kim et al., 2015). (B) *Alexandrium fracterculus* (Lee et al., 2019). (C) *Fragilaria crotonensis* (Michel et al., 2006). (D) *Tetracyclus glans* (Michel et al., 2006). I *Cyclotella quillensis* (Saros and Fritz, 2000). (F) *Cymbella pusilla* (Saros and Fritz, 2000). (G) *Anomoeoneis costata* (Saros and Fritz, 2000). (H) *Microcystis aeruginosa* (Sugimoto et al., 2015).

The relationship between phytoplankton growth rate and nutrient supply is theoretically described by Monod kinetics. The Monod equation is a mathematical kinetic model to describe specific microbial growth rate (μ) as a function of substrate concentrations following Eq. (1), (Monod, 1949).

(1) $$\mu =\;\mu_{max}\frac{S}{K_{s}+S}$$

where $\mu_{max}$ is the maximum specific growth rate (day^−1^) of microorganisms at substrate saturation, *S* is the substrate concentration (*µ*M), and *K_S_* is the half-saturation constant (*µ*M) as a value of substrate concentration corresponding to half of *μ_max_*. The equation indicates that the growth rate of phytoplankton is variable based on nutrient supply, which is seen as an essential and limiting substrate. Subsequent studies have evaluated Monod's theory with various bacteria and substrates, showing the similarity to Monod kinetics on bacterial growth rate while suggesting several modifications to this theory (Koch, 1982; Owens and Legan, 1987; Shehata and Marr, 1971). Today, the Monod formulation is still widely used to model the relationship between growth rate and nutrient supply (Fig. 1, Fig S1).

The downside of Monod kinetics is that it carries limited information in cellular physiology. It imposes the maximum growth rate that fits the data, but the equation does not inform us what controls this maximum growth rate. Also, whereas the saturating equation can be fitted to most of the data, why it produces such a saturating relationship between the growth rate and nutrient concentration remains elusive. Thus, the scientific community desires a more physiologically defensible model (Follows and Dutkiewicz, 2011). Recently, a study of heterotrophic bacteria implies that the high-nutrient-end growth limitation may partly be caused by some intracellular effect (Casey and Follows, 2020). Here, we focus on phytoplankton species and explore what intracellular factors constrain the growth rate.

A mechanistic model (i.e., Cell Flux Model of Phytoplankton, CFM-Phyto) was recently developed which outputs the relationship between growth rate, elemental stoichiometry, and macromolecular allocations (e.g., proteins, DNA, RNA, carbohydrates, and chlorophyll) in phytoplankton given different environmental conditions. Initial environmental parameters which can be used include varying nutrient regimes, temperature, and light intensity (Armin and Inomura, 2021; Inomura et al., 2020). CFM-Phyto has been shown to well capture the observed trends of elemental stoichiometry of various phytoplankton (Chalup and Laws, 1990; Healey, 1985; Inomura et al., 2020; Sakshaug and Andersen, 1989), supporting its structural robustness. The model has been used for various purposes, such as predicting C:P ratios in the ocean based on the satellite remote sensing data (Tanioka et al., 2020) and light and temperature dependencies of C:N:P ratios (Armin and Inomura, 2021; Inomura et al., 2020). Accordingly, CFM-Phyto demonstrated again that phytoplankton are critical components in the ocean and have a significant impact on global primary production, biogeochemical cycles, and marine ecosystems. Not only this, but CFM-Phyto also provided key insights to cellular physiology under varying nutrient conditions (e.g., nitrogen limitation and phosphorus limitation), temperature (Armin and Inomura, 2021), and light intensity (Inomura et al., 2020). Furthermore, CFM-Phyto continually demonstrates that the model is capable of accurately capturing realistic trends consistent with observations.

In this study, we adapt CFM-Phyto (Inomura et al., 2020) and link nutrient uptake and macromolecular allocation to interpret the saturating relationship between the growth rate and nutrient concentration. We developed the model to address the following questions: (1) Can CFM-Phyto represent data as accurately as Monod Kinetics? (2) What leads to the saturating relationship between the growth rate and nutrient concentration? Here, we focus on the relationship between the growth rate and the concentration of one of the major nutrients, NO_3_^−^, using data of phytoplankton across taxa. Our model-data comparison emphasizes the strength of the model, accurately representing multiple datasets, and suggests the Monod kinetics model is not the only model which can be utilized data such as these. Moreover, the model provides a macromolecular-based interpretation of this widely observed saturating relationship, expanding on the knowledge offered by previously created models.

## 2 Methods

Here, we describe how we optimally modeled observations of phytoplankton- NO_3_^−^ interactions first using the Monod equation, then a cell flux model of phytoplankton (CFM-Phyto) to investigate cellular processes typically described by Monod kinetics. We used data from published papers with 12 species of phytoplankton inlcuding *Alexandrium affine, Alexandrium fracterculus, Anomoeoneis costata, Asterionella formosa, Cyclotella quillensis, Cyclotella* sp*, Cymbella pusilla, Fragilaria crotonensis, Microcystis aeruginosa, Staurosirella pinnata, Synechocystis* sp. PCC6803*,* and *Tetracyclus glans* under nitrogen limited conditions (Kim et al., 2015; Lee et al., 2019; Michel et al., 2006; Saros and Fritz, 2000; Sugimoto et al., 2015) (Table S1). Together, these provided a large range of phytoplankton taxa grown under NO_3_^−^ limited conditions to test with both methods.

### 2.1 Monod-kinetics

Once we selected data, we optimized the Monod kinetics curve Eq. (1) for each dataset using a Markov Chain Monte Carlo (MCMC) method, specifically, the Metropolis-Hastings algorithm (Hastings, 1970; Metropolis et al., 1953; Omta et al., 2017). This algorithm is an iterative numerical method that introduces perturbations to our initial estimates and eventually converges to parameter values that best fit the data. We assessed the optimization results produced by the algorithm with visual trial-and-error, changing our initial estimates for the maximum growth rate ($\mu_{max}$) and the half-saturation constant ($K_{s}$) when necessary. For each dataset, we recorded the best values for the maximum growth rate and the half-saturation constant (Table S2). The results are in Fig. 1.

### 2.2 CFM representation

Next, we used CFM-Phyto to model the relationship seen in the data. CFM-Phyto is a coarse-grained model that predicts macromolecular allocation of nutrients (here we focus on C and N) to major pools of biological molecules and the resulting cellular elemental stoichiometry under various environmental conditions (Inomura et al., 2020). In the supplemental material, we provided a simple flowchart that illustrates how the model runs (Fig. S2). Key assumptions of the model include linear relationships between the RNA, protein, and growth rate (Jahn et al., 2018; Nicklisch and Steinberg, 2009; Scott et al., 2010; Zavrel et al., 2019), a constant macromolecular composition of the photosynthetic machinery (Folea et al., 2008; Geider and MacIntyre, 1996; Kirchhoff, 2014; Kirchhoff et al., 2008), and a saturating function between irradiance and photosynthesis (Cullen, 1990; Geider, 1998).

**Fig. 2:**
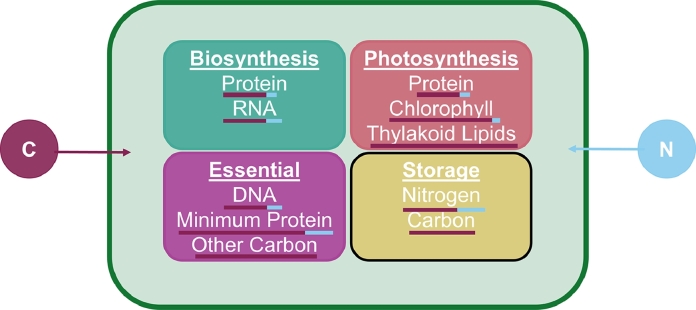
The CFM allocates C (maroon) and N (blue) to 4 intracellular macromolecular pools: biosynthesis (teal), photosynthesis (pink), essential (purple), and storage (yellow). Each pool contains different macromolecules with varying C and N allocated to each (Liefer et al., 2019), indicated by the bar below the macromolecule. Storage changes based on which nutrient is limiting, marked by the black outline around this box. When N is limited, there is no allocation of N or C to N storage. Similarly, when C is limited, there is no allocation of C to C storage. Essential macromolecules remain constant throughout simulations as they represent macromolecules needed for basic cell survival and structure.

Here, we grouped biomolecules into 4 categories: photosynthesis, biosynthesis, essential, and storage (Fig. 2). Photosynthetic macromolecules include proteins, chlorophyll, and lipids in the thylakoid membranes, biosynthetic macromolecules include protein and RNA, and essential macromolecules are molecules necessary for basic cell survival and cell structure such as DNA, a minimum level of protein, and other C. Storage is only available when excess nutrients are available. Here, we ran the model in N limitation and C limitation. Thus, C storage only occurs when C is not limited and likewise, N storage occurs when N is not limited.

Several key equations informed the macromolecular allocation within the model. For an extensive list of all equations, parameters, their respective definitions, and derivations please refer to Table S3 and S4 in the supplementary material. Some noteworthy equations Eq. (2)-Eq. (8) describe the overview of macromolecular allocation of C and N as well as the solution for growth rate under C and N limitations. We defined 8 categories in which C is allocated to within our model and, as an approximation, assumed that these defined pools comprise all C within the cell, which is represented by the sum of all C pools equated to 1 Eq. (2).

(2) $$1=Q_{C}^{Pro}+Q_{C}^{RNA}+Q_{C}^{DNA}+Q_{C}^{Chl}+Q_{C}^{Plip-Thy}+Q_{C}^{Nsto}+Q_{C}^{Csto}+Q_{C}^{Oth}$$

The categories we used include proteins ($Q_{C}^{Pro}$), RNA molecules ($Q_{C}^{RNA}$), DNA molecules ($Q_{C}^{DNA}$), chlorophyll ($Q_{C}^{Chl}$), phospholipids in the thylakoid membranes ($Q_{C}^{Plip-Thy}$), N storage ($Q_{C}^{Nsto}$), C storage ($Q_{C}^{Csto}$), and all remaining C labeled as other ($Q_{C}^{Oth}$). This equation with the above key assumption leads to a quadratic equation (See Table S3 for derivation):

(3) $$0=a_{C}\mu^{2}+b_{C}\mu +c_{C}$$

and the solution for μ provides the growth rate based on C allocation (or C limitation). A suite of parameters from previously described biomolecule definitions (Inomura et al., 2020) remake up the terms $a_{C}$, $b_{C}$, $c_{C}$, and (see Table S3 for details).

To consider N limitation, we describe the change of cellular N concentration over time ($\frac{dQ_{N}}{dt}$) by subtracting the N dedicated to new cell growth ($\mu Q_{N}$) from the rate of N uptake ($V_{N}$).

(4) $$\frac{dQ_{N}}{dt}=V_{N}-\mu Q_{N}$$

We assumed steady-state conditions, meaning there is no change in the cellular N concentration over time ($\frac{dQ_{N}}{dt}=0)$ and Eq. (4) becomes

(5) $$V_{N}=\mu Q_{N}$$

where the cellular N is defined by the macromolecular allocation of N Eq. (7). This equation assumes that the cellular N uptake is limited by the rate of diffusion; thus, the uptake is proportional to NO_3_^−^ concentration (Casey and Follows, 2020):

(6) $$V_{N}=A_{N}\left[NO_{3}^{-}\right]$$

where *A_N_* is a constant value. In Eq. (5), *Q_N_* is represented by the combination of macromolecules that contains N (Inomura et al., 2020):

(7) $$Q_{N}=Q_{N}^{Pro}+Q_{N}^{RNA}+Q_{N}^{DNA}+Q_{N}^{Chl}+Q_{N}^{Nsto}$$

The model allocates N to proteins ($Q_{N}^{Pro}$), RNA molecules ($Q_{N}^{RNA}$), DNA molecules ($Q_{N}^{DNA}$), chlorophyll ($Q_{N}^{Chl}$), and N storage ($Q_{N}^{Nsto}$). Similarly to C allocation, with the key assumptions above, we may rearrange this equation, which leads to the following cubic relationship (see Table S3 for derivation):

(8) $$0=a_{N}\mu^{3}+b_{N}\mu^{2}+c_{N}\mu +d_{N}$$

A suite of parameters from previously described biomolecule (Inomura et al., 2020) definitions make up the terms $a_{N}$, $b_{N}$, $c_{N}$, and $d_{N}$ (Table S3). Here, the major difference between the two equations is that solving for N requires a cubic, rather than a quadratic, function. This occurs due to the additional growth rate factor Eq. (5) to balance the uptake rate of N.

Lastly, we parameterized the model to match the light intensity of each experiment and made initial estimates on the mass ratio for the cellular photosynthetic proteins to chlorophyll ratio (*A_pho_*) and the affinity to nitrate (*A_N_*). To keep the problem simple, we assumed a constant ratio between *A_pho_* and the mass of biosynthetic protein based on default run of the previous CFM-Phyto (Inomura et al., 2020). Again, we used the Metropolis-Hastings algorithm to converge to the best representation of the data. As we did for the Monod optimization procedure, the algorithm predicted the best values for *A_pho_* and *A_N_* (Table S5).

## 3 Results and discussion

**Fig. 3:**
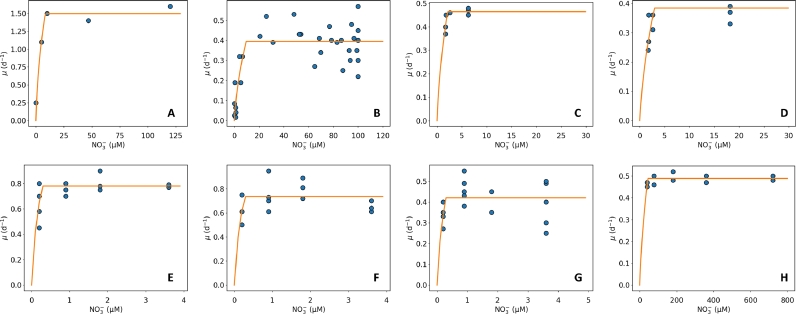
CFM-Phyto representation of Growth rate vs NO3- concentration. Dots are Data and Curves are model results with CFM-Phyto (Curves). (A) *Synechocystis* sp. PCC6803 (Kim et al., 2015). (B) *Alexandrium fracterculus* (Lee et al., 2019). (C) *Fragilaria crotonensis* (Michel et al., 2006). (D) *Tetracyclus glans* (Michel et al., 2006). (E) *Cyclotella quillensis* (Saros and Fritz, 2000). (F) *Cymbella pusilla* (Saros and Fritz, 2000). (G) *Anomoeoneis costata* (Saros and Fritz, 2000). (H) *Microcystis aeruginosa* (Sugimoto et al., 2015).

We tested the CFM-Phyto with the data of growth vs. NO_3_^−^ concentrations for 12 species including *Alexandrium affine, Alexandrium fracterculus, Anomoeoneis costata, Asterionella formosa, Cyclotella quillensis, Cyclotella* sp*, Cymbella pusilla, Fragilaria crotonensis, Microcystis aeruginosa, Staurosirella pinnata, Synechocystis* sp. PCC6803*,* and *Tetracyclus glans* under nitrogen limited conditions (Kim et al., 2015; Lee et al., 2019; Michel et al., 2006; Saros and Fritz, 2000; Sugimoto et al., 2015) (Table S1, Fig. 3, Fig. S3). Overall, the CFM-Phyto shows a similar pattern as the Monod mathematical model (compare Fig. 1 and Fig. 3), capturing the overall pattern of the data with two components: an increasing part and a stable part. This trend is clear in the data and, qualitatively, CFM-Phyto may represent the pattern even more accurately than Monod kinetics, since the latter imposes a continuously increasing growth rate with nitrate concentration ([NO_3_^−^]), which is not true for many data; there is not a clear continued increase in most of the data. However, at high [NO_3_^−^], the increase in the growth rate is minimal in Monod kinetics, thus, these two different models are similar and almost equally capturing the observed trends.

However, whereas the Monod kinetics formulation is an elegant model with minimum parameters, it carries little information of cellular physiology, having left the physiological mechanisms vague. It may provide information about nutrient uptake, yet these nutrients must be processed internally to make cellular materials, and the Monod kinetics is a black box regarding the internal processes.

**Fig. 4:**
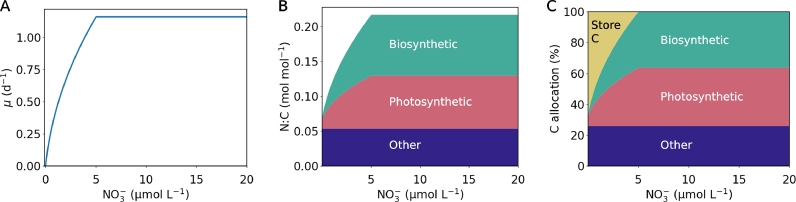
Model results of growth rate, cellular N:C, and macromolecular allocation. (A) Growth rate. (B) Cellular N:C ratio and macromolecular allocation. (C) C allocation in%.

CFM-Phyto, on the other hand, provides data-backed physiological insights into a commonly observed pattern of the growth-nutrient relationship (Inomura et al., 2020). The increasing growth rate is accompanied by increasing photosynthetic and biosynthetic molecules (Fig. 4A,B) because a higher growth rate requires a higher amount of the cellular building apparatus. Specifically, the cell requires more photosynthetic molecules for providing fixed C and more biosynthetic molecules to process fixed C and other nutrients to build terminal cellular materials. The increased investment in such a cellular building apparatus results in increased N:C because most of the mass in this apparatus consists of protein, which has high N:C ratios (∼1:4) (Geider and Roche, 2002; Inomura et al., 2020). This trend is supported by laboratory studies where allocation to proteins increase with the growth rate as well as N:C of the cells (Felcmanova et al., 2017; Liefer et al., 2019). When [NO_3_^−^] is small, the cell accumulates C storage, keeping the N:C ratio low, but the storage decreases with [NO_3_^−^], and is replaced by biosynthetic and photosynthetic molecules. When the cell transitions from N limitation to C limitation, the growth rate does not increase with [NO_3_^−^] since, the cell uses its full capacity to allocate C to the biosynthetic and photosynthetic molecules with little or no dedication to C storage molecules (Fig. 4). These macromolecular interpretations are consistent with the general pattern of macromolecular allocation (Felcmanova et al., 2017; Jahn et al., 2018) and elemental stoichiometry (Chalup and Laws, 1990; Healey, 1985; Sakshaug and Andersen, 1989).

**Fig. 5:**
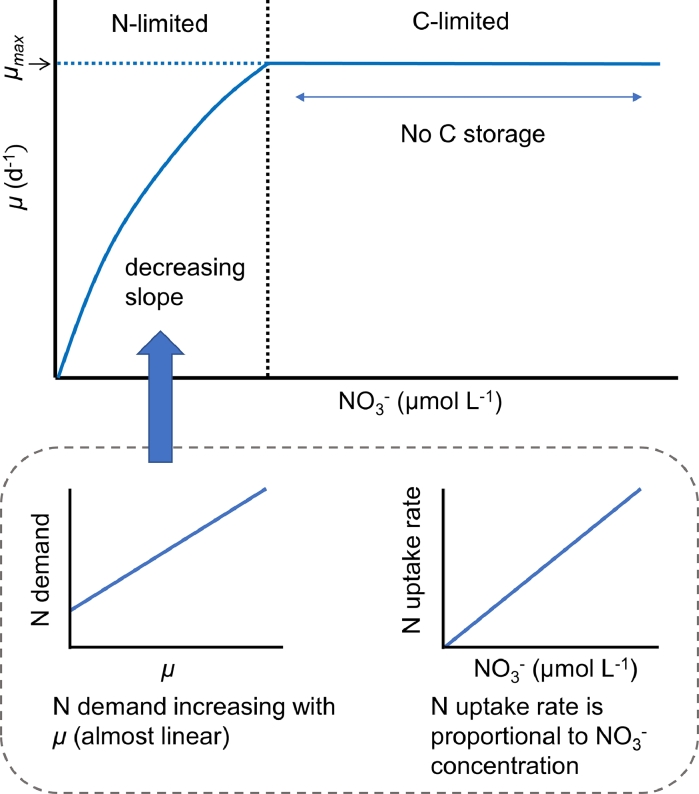
Summary of this study. Monod kinetics may be explained by the combination of two parts. N-limited (with low NO_3_^−^) and C-limited (with high NO_3_^−^). Here we consider N demand and N uptake rate per cellular C. Under N limitation, we may expect linear increase of growth with NO_3_^−^ concentration, but increasing N demand for growth and photosynthetic machineries creates non-linearity in *μ* vs NO_3_^−^ relationship. When it reaches C-limitation, there is no more C storage to be allocated to growth and photosynthetic machineries; thus, the growth rate does not increase even with increased NO_3_^−^.

The capability of CFM-Phyto to capture the growth-NO_3_^−^ data (Fig. 3) suggests that there are two phases depending on NO_3_^−^concentrations: N limitation and C limitation (Fig. 5). Under N limitation, uptake of NO_3_^−^ is balanced by the ‘loss’ of N to new cells (growth) as in Eq. (5). Rearranging the equations tells us that the growth rate is represented by N uptake per cellular quota of N:

(9) $$\mu =\frac{V_{N}}{Q_{N}}$$

Since intracellular N (relative to C) increases with the growth rate (Fig. 4B), given the N uptake (relative to C) rate is proportional to the NO_3_^−^ concentration, the slope of the μ-NO_3_^−^ relationship decreases (Fig. 4B). This effect leads to a decreased slope of μ-NO_3_^−^ relationship (Fig. 4A, Fig. 5), as can be seen in data and Monod kinetics. As NO_3_^−^ concentration increases, the N quota reaches a maximum with highest allocation to growth-related molecules because, at this point, no more C is left for additional growth-related molecules (C limitation). The model predicts that the transition between N and C limitation results in rather abrupt slope change, the trend that is shown across data (Fig. 3, Fig. S3).

In this study, we focused on the growth rate dependences on NO_3_^−^ concentration. Intracellular allocation of other elements such as phosphorus (P) can be affected because it is a part of biosynthetic and photosynthetic molecules (Inomura et al., 2020; Liefer et al., 2019; Rhee, 1978). However, cellular P:C may be rather stable because the amount of phosphorus storage functions as a buffer for the totally cellular P per C (Inomura et al., 2020). Under phosphorus limitation, CFM-Phyto predicts non-linear increase in P:C (Inomura et al., 2020), which are backed by data (Elrifi and Turpin, 1985; Garcia et al., 2016; Healey, 1985). This may affect the model result differently than N limited case because the N:C increase linearly with the growth rate. However, given the growth dependencies of macromolecular allocations that is rich in P (e.g., phospholipid and RNA), we predict that macromolecular allocation is also an important factor in growth rate-phosphorus relationship. There are other factors that influences the growth of phytoplankton, such as light (Inomura et al., 2020; Thompson et al., 1989), temperature (Eppley, 1972), pH (Abinandan et al., 2021), and Fe (Sunda and Huntsman, 1995), etc. Our model may provide a useful framework for further investigating both independent and dependent limitations of these factors.

The model assumes a constant elemental composition of elemental stoichiometry within macromolecules (e.g., C:N in proteins is 4.49:1) (Inomura et al., 2020). As above, we assume a constant composition of photosynthetic machinery, whereas in reality it can vary. We make these simplifications for two reasons. 1) There simplification allows keeping the number of free parameters low; increasing the level of details may lead to more unconstrained parameters. 2) We may not have enough data to generalize these variations. Despite such a simplification, our model may well capture the elemental stoichiometry across taxa (Inomura et al., 2020), which may suggest that the factors that we simplified have only secondary effects. We note that despite these simplifications, our model resolves more detailed macromolecular allocations than widely used models (e.g., Droop types), and thus these simplifications are done at more detailed levels (Armin and Inomura, 2021; Inomura et al., 2020) than widely used models, including Monod kinetics (Monod, 1949). Further experiments must be performed for the incorporation of further details.

In this study, we focused on two guiding questions: (1) Can CFM-Phyto represent data as accurately as Monod Kinetics? (2) What leads to the saturating relationship between the growth rate and nutrient concentration? We found that in most cases, CFM-Phyto represents data comparably with Monod kinetics, but in a few instances CFM-Phyto captures the trend more accurately. Additionally, the saturating relationship between growth rate and nutrient concentration may be explained by the combination of N-limitation and C-limitation within the cell. Under N limitation, the increasing N demand for growth and photosynthetic machineries creates non-linearity in *μ* vs NO_3_^−^ relationship. When the cell reaches C-limitation, there is no more C in storage that can be allocated to growth and photosynthetic machineries; thus, the growth rate does not increase even with increased [NO_3_^−^] leading to the observed saturated trend.

## 4 Conclusion

Overall, CFM-Phyto produces a general relationship between *μ* and NO_3_^−^, represents data from various taxa, and provides a macromolecular interpretation of how NO_3_^−^ gradually saturates, often modeled by Monod kinetics. CFM-Phyto thus provides a useful tool for representing cellular growth of phytoplankton, simulating their growth in culture systems and nature including lakes and the ocean. As opposed to Monod kinetics and other models focused on nutrient uptake, our study suggests that internal processes and molecular allocation plays an important role in constraining the nutrient vs growth relationship. Not only did we capture similar trends using a different method, but we were also able to provides more insight about the key cellular processes that lead to this commonly observed trend that are often lacking in conventional models. This work offers a new toolkit that considers cellular physiology to improve representation of the relationship between nutrients and growth. Ultimately, this model captures realistic trends which is exciting and promising for future incorporation into large ecosystem and biogeochemical models.

## Author contributions

All the authors conceived and designed this study, collected data, ran the model, and wrote the manuscript. All authors equally contributed to this study.

## Model availability

The code for the CFM-Phyto used in this study can be found here: https://zenodo.org/record/6407154 (DOI: 10.5281/zenodo.6407154).

## Declaration of Competing Interest

The authors declare that they have no known competing financial interests or personal relationships that could have appeared to influence the work reported in this paper.

## Appendix Supplementary materials

Image, application 1
