# Supplementary material for: Saturating relationship between phytoplankton growth rate and nutrient concentration explained by macromolecular allocation
Source: Curr Res Microb Sci. 2022 Sep 21;3:100167. doi: 10.1016/j.crmicr.2022.100167 (PMC9742995; doi:10.1016/j.crmicr.2022.100167)
Supplement: Supplementary file 1 [file mmc1.pdf]

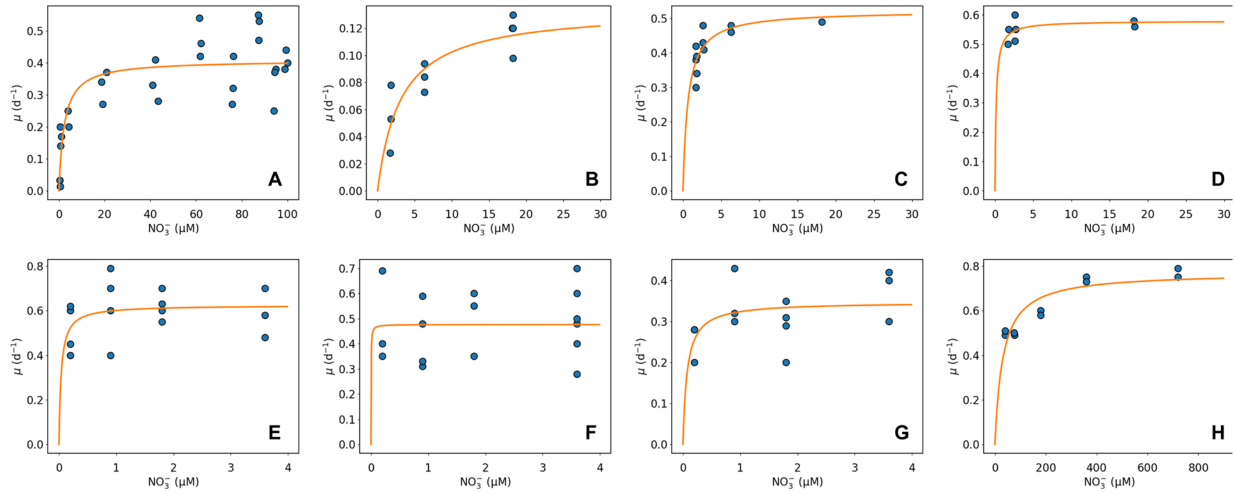

Fig. S1. Monod representation of growth rate vs NO^{3-} concentration. Dots are data and curves are Monod formulation. (A) *Alexandrium affine* (Lee et al., 2019). (B) *Asterionella Formosa* (Michel et al., 2006). (C) *Fragilaria crotonensis* (Michel et al., 2006). (D) *Staurosirella pinnata* (Michel et al., 2006). (E) *Cyclotella quillensis* (Saros and Fritz, 2000). (F) *Cymbella pusilla* (Saros and Fritz, 2000). (G) *Anomoeoneis costata* (Saros and Fritz, 2000). (H) *Cyclotella* sp (Sugimoto et al., 2015).

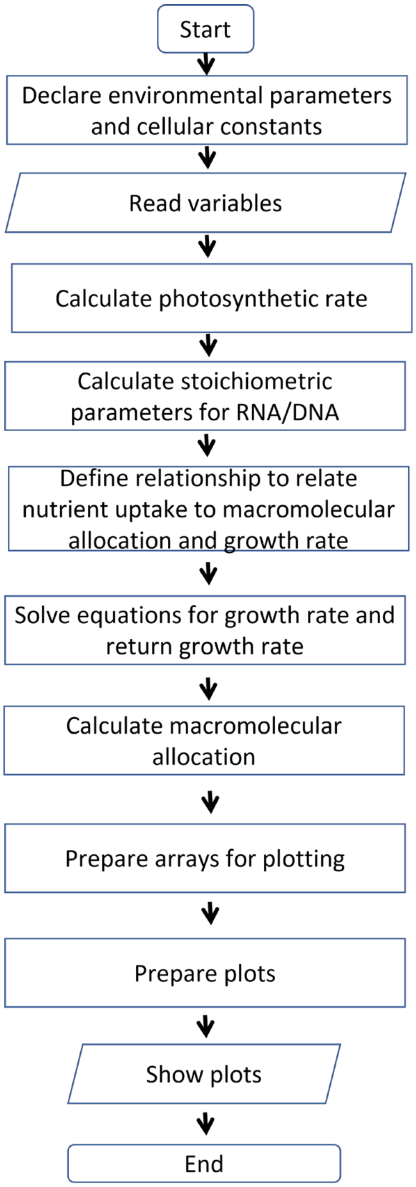

Fig. S2. Basic flowchart for model code. Here, rounded rectangles signify terminal points in the code, rectangles represent processing steps, and the rhombuses show input or output.

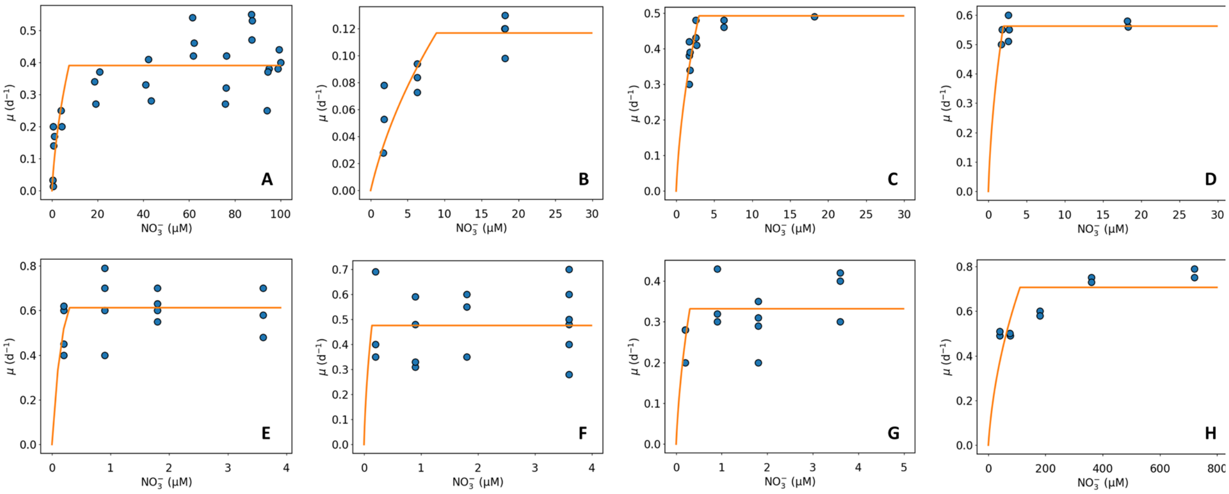

Fig. S3. CFM-Phyto representation of growth rate vs NO^{3-} concentration. Dots are data and curves are model results. (A) *Alexandrium affine* (Lee et al., 2019). (B) *Asterionella Formosa* (Michel et al., 2006). (C) *Fragilaria crotonensis* (Michel et al., 2006). (D) *Staurosirella pinnata* (Michel et al., 2006). (E) *Cyclotella quillensis* (Saros and Fritz, 2000). (F) *Cymbella pusilla* (Saros and Fritz, 2000). (G) *Anomoeoneis costata* (Saros and Fritz, 2000). (H) *Cyclotella* sp (Sugimoto et al., 2015).

Table S1. Sources of datasets with key details from the experimental set-up used to inform our model and the Monod mathematical model.

Source	Phytoplankton	Light intensity reported	Nutrient information	Note
(Lee et al., 2019)	*Alexandrium affine, Alexandrium fracterculus*	100 (μmol photons m^{-2} s^{-1})	110 μM nitrogen	Nutrient replete
(Michel et al., 2006)	*Asterionella Formosa, Fragilaria crotonensis, Staurosirella pinnata, Tetracyclus glans*	1170, 878, 527 (μmol photons m^{-2} s^{-1})	P = 5 μM, N = 18 μM.	Differing light intensities Nutrients added in excess unless it was limiting then concentration to right used.
(Saros and Fritz, 2000)	*Cyclotella quillensis, Cymbella pusilla,*	45 (μmol photons m^{-2} s^{-1})	Concentrations of 0.225, 0.9, 1.8, 3.6 and 18 μM nitrogen.	Used a different range of salinities 5, 8, 11 ppt

	Anomoeoneis costata			
(Sugimoto et al., 2015)	*Cyclotella* sp, *Microcystis aeruginosa*	135 (μmol photons m^{-2} s^{-1})	The nitrate concentration in the medium was initially set at 35.7, 71.4, 178, 357, and 714 μM.	
(Kim et al., 2015)	*Synechocystis* sp. PCC6803		N ≥ 2.3 mgN/L, P ≥ 0.1 mgP/L, and C ≥ 1.0 mgC/L.	

Table S2. Resultant maximum growth rates (μ_{max}), half-saturation constants (K_S) from Monod kinetics optimization.

Figure Number	μ_{max}	K_S
1A	1.991822071	1.670832957
1B	0.429965472	2.830548571
1C	0.571786541	0.157563929
1D	0.402053583	0.469228183
1E	0.790089622	0.050585305
1F	0.807350406	0.035802264
1G	0.420666096	0.042858369
1H	0.515527146	2.963413845
S1A	0.518895477	2.283033689
S1B	0.137588831	3.100046879
S1C	0.488421201	0.672924588
S1D	0.571786541	0.157563929
S1E	0.626918484	0.040216599
S1F	0.498324079	0.002907321
S1G	0.360033608	0.070640680
S1H	0.858109278	31.15261906

Table S3. List of equations used in CFM.

$Q_C^{Plip-Thy} = Q_P^{Thy} Y_{Plip}^{C:P}$	1
$Q_P^{Thy} = A_{Pho}^{P:Chl} Q_C^{Chl}$	2
$Q_C^{Chl} = A_{Chl}(I)\mu + B_{Chl}(I)$	3

$$A_{Chl}(I) = \frac{(1+E)}{v_I(I)}$$	4
$$B_{Chl}(I) = \frac{m}{v_I(I)}$$	5
$$Q_C^{RNA} = Q_P^{RNA} Y_{RNA}^{C:P}$$	6
$$Q_P^{RNA} = A_P^{RNA} \mu Q_C^{Pro} + Q_{P,min}^{RNA}$$	7
$$Q_C^{Pro} = Q_C^{Pro-bio} + Q_C^{Pro-pho} + Q_C^{Pro-oth}$$	8
$$Q_C^{Pro-bio} = A_{bio}\mu$$	9
$$Q_C^{Pro-pho} = A_{pho} Q_C^{Chl}$$	10
$$1 = Q_C^{Pro-bio} + Q_C^{Pro-pho} + Q_C^{Pro-oth} + Q_C^{RNA} + Q_C^{DNA} + Q_C^{Chl} + Q_C^{Plip-Thy} + Q_C^{Nsto} + Q_C^{Csto} + Q_C^{Oth}$$	11
$$Q_N = Q_N^{Pro} + Q_N^{RNA} + Q_N^{DNA} + Q_N^{Chl}$$	12
$$Q_N = Y_{Pro}^{N:C} Q_N^{Pro} + Y_{RNA}^{N:C} Q_N^{RNA} + Y_{DNA}^{N:C} Q_N^{DNA} + Y_{Chl}^{N:C} Q_N^{Chl}$$	13
$$V_N = \mu Q_N$$	14
$$0 = a_C\mu^2 + b_C\mu + c_C$$ where $$a_C = Y_{RNA}^{C:P} A_{RNA}^{P} (A_{pho} A_{Chl}(I) + A_{bio})$$ $$b_C = \left(1 + A_{pho} + Y_{Plip}^{C:P} A_{Pho}^{P:Chl}\right) A_{Chl}(I) + A_{bio} + Y_{RNA}^{C:P} A_{RNA}^{P} (A_{pho} B_{Chl}(I) + Q_C^{Pro-oth})$$ $$c_C = \left(1 + A_{pho} + Y_{Plip}^{C:P} A_{Pho}^{P:Chl}\right) B_{Chl}(I) + Q_C^{Oth} + Y_{RNA}^{C:P} Q_{P,min}^{RNA} - 1$$	15
$$a_N\mu^3 + b_N\mu^2 + c_N\mu + d_N$$ where $$a_N = Y_{RNA}^{N:P} A_{RNA}^{P} (A_{pho} A_{Chl}(I) + A_{bio})$$ $$b_N = Y_{Chl}^{N:C} A_{Chl}(I) + Y_{Pro}^{N:C} \left(A_{bio} + A_{pho} A_{Chl}(I)\right) + Y_{RNA}^{N:P} A_{RNA}^{P} (A_{pho} B_{Chl}(I) + Q_C^{Pro-Oth})$$ $$c_N = Y_{Chl}^{N:C} B_{Chl}(I) + Y_{Pro}^{N:C} \left(Q_C^{Pro-Oth} + A_{pho} B_{Chl}(I)\right) + Y_{RNA}^{N:P} Q_{P,min}^{RNA} + Y_{DNA}^{N:C} Q_C^{DNA}$$ $$d_N = -V_N$$	16

*Usually, Q_N has the added parameter, Q_N^{Nsto}, but is excluded from our equation to simulate nitrogen limitation here.

Table S4. List of definitions and units of measurement for parameters used in CFM equations.

Parameter	Definition	Units
$Q_C^{Plip-Thy}$	Amount of carbon dedicated to the phospholipids in the thylakoid membranes	mol C mol C^{-1}
Q_P^{Thy}	Amount of phosphorus in thylakoid membranes	mol P mol C^{-1}
$Y_{Plip}^{C:P}$	Stoichiometric ratio carbon to phosphorus in	mol C (mol P in Phospholipid membrane)$^{-1}$

	phospholipids in thylakoid membrane	
$A_{Pho}^{P:Chl}$	Constant of proportionality relating chlorophyll to the thylakoid membranes	mol P mol C^{-1}
Q_C^{Chl}	Amount of carbon in chlorophyll	mol C mol C^{-1}
$A_{Chl}(I)$	Term to represent photosynthetic rate	
$B_{Chl}(I)$	Term to represent cell maintenance	
μ	Growth rate	d^{-1}
E	Respiration rate	
v_I	Photosynthetic rate	mol C (mol C in Chl)$^{-1}$ d^{-1}
I	Irradiance	µmol photons m^{-2} s^{-1}
m	Cell maintenance rate	d^{-1}
Q_C^{RNA}	Amount of carbon in RNA molecules	mol C mol C^{-1}
Q_P^{RNA}	Amount of phosphorus in RNA molecules	mol P mol C^{-1}
$Y_{RNA}^{C:P}$	Stoichiometric ratio carbon to phosphorus in RNA molecules	mol C (mol P in RNA)$^{-1}$
A_P^{RNA}	Constant of proportionality relating protein, growth rate and RNA molecules	mol P mol C^{-1}d
Q_C^{Pro}	Amount of carbon in proteins	mol C mol C^{-1}
$Q_{P,min}^{RNA}$	The minimum amount of phosphorus in RNA molecules	mol P mol C^{-1}
$Q_C^{Pro-bio}$	Amount of carbon in biosynthetic proteins	mol C mol C^{-1}
$Q_C^{Pro-pho}$	Amount of carbon in photosynthetic proteins	mol C mol C^{-1}
$Q_C^{Pro-oth}$	Other carbon in proteins	mol C mol C^{-1}
A_{bio}	Constant of proportionality relating growth rate and biosynthetic protein	mol C mol C^{-1}d
A_{pho}	Constant of proportionality relating chlorophyll and photosynthetic protein	mol C (mol C in Chl)$^{-1}$ d^{-1}
Q_C^{DNA}	Amount of carbon in DNA molecules	mol C mol C^{-1}

Q_C^{Nsto}	Amount of carbon in nitrogen storage	mol C mol C^{-1}
Q_C^{Csto}	Amount of carbon in carbon storage	mol C mol C^{-1}
Q_C^{Oth}	Amount of leftover carbon within the cell	mol C mol C^{-1}
Q_N	Amount of cellular nitrogen	mol N cell^{-1}
Q_N^{Pro}	Amount of nitrogen in proteins	mol N mol C^{-1}
Q_N^{RNA}	Amount of nitrogen in RNA molecules	mol N mol C^{-1}
Q_N^{Chl}	Amount of nitrogen in chlorophyll	mol N mol C^{-1}
Q_N^{DNA}	Amount of nitrogen in DNA molecules	mol N mol C^{-1}
$Y_{Pro}^{N:C}$	Stoichiometric ratio of nitrogen to carbon in protein	mol N (mol C in protein)$^{-1}$
$Y_{RNA}^{N:C}$	Stoichiometric ratio of nitrogen to carbon in RNA	mol N (mol C in RNA)$^{-1}$
$Y_{DNA}^{N:C}$	Stoichiometric ratio of nitrogen to carbon in DNA	mol N (mol C in DNA)$^{-1}$
$Y_{Chl}^{N:C}$	Stoichiometric ratio of nitrogen to carbon within chlorophyll	mol N (mol C in Chl)$^{-1}$
V_N	Uptake rate of nitrogen into the cell	mol N cell^{-1}d^{-1}

To obtain the above quadratic and cubic functions, first it is necessary to substitute of all parameters, so no equation goes unused. To get the carbon equation, use equations 1-10 to substitute parameters in equation 11. If there are no parameters to substitute for a given variable (i.e. Q_C^{DNA}) then the parameter is a constant and remains as is in the equation. Once this has been accomplished, combine like terms and group them based on growth rate. This will give you the above quadratic equation. For nitrogen, use equations 12 and 13 and substitute into equation 14. Again, once this is done, group alike terms and simplify. This will give the above cubic formation.

Table S5. Resultant (A_{pho}), (A_N), from CFM-Phyto optimization.

Figure Number	A_{pho}	A_N
3A	3.396970363	2.70E-20
3B	2.875243021	1.08E-20
3C	9.999182639	3.53E-20
3D	13.28046614	3.11E-20
3E	2.009096435	5.61E-19
3F	2.128989742	5.46E-19
3G	2.244261632	1.32e-21
3H	9.983819725	3.46e-20

S2A	2.875243021	1.08E-20
S2B	29.70651438	2.69E-21
S2C	9.999182639	3.53E-20
S2D	29.66777302	2.77e-21
S2E	2.007996775	5.60E-19
S2F	3.181372549	5.85E-19
S2G	9.377358325	2.30e-19
S2H	2.201161315	1.43E-21

References

Kim, H.-W., Park, S., Rittmann, B.E., 2015. Multi-component kinetics for the growth of the cyanobacterium *Synechocystis* sp. PCC6803. Environ Eng Res 20(4), 347-355.

Lee, K.H., Jeong, H.J., Kang, H.C., Ok, J.H., You, J.H., Park, S.A., 2019. Growth rates and nitrate uptake of co-occurring red-tide dinoflagellates *Alexandrium affine* and *A. fraterculus* as a function of nitrate concentration under light-dark and continuous light conditions. Algae 34(3), 237-251.

Michel, T.J., Saros, J.E., Interlandi, S.J., Wolfe, A.P., 2006. Resource requirements of four freshwater diatom taxa determined by in situ growth bioassays using natural populations from alpine lakes. Hydrobiologia 568(1), 235-243.

Saros, J.E., Fritz, S.C., 2000. Changes in the growth rates of saline-lake diatoms in response to variation in salinity, brine type and nitrogen form. J Plankton Res 22(6), 1071-1083.

Sugimoto, K., Negishi, Y., Amano, Y., Machida, M., Imazeki, F., 2015. Roles of dilution rate and nitrogen concentration in competition between the cyanobacterium *Microcystis aeruginosa* and the diatom *Cyclotella* sp. in eutrophic lakes. J Appl Phycol 28(4), 2255-2263.
